# Supplementary material for: Efficient photon-pair generation in layer-poled lithium niobate nanophotonic waveguides
Source: Light Sci Appl. 2024 Oct 3;13:282. doi: 10.1038/s41377-024-01645-5 (PMC11450222; doi:10.1038/s41377-024-01645-5)
Supplement: Supplementary file 1 — Supplementary Information [file 41377_2024_1645_MOESM1_ESM.pdf]

Supplementary Information for Efficient photon-pair generation in layer-poled lithium niobate nanophotonic waveguides

Xiaodong Shi[1,†], Sakthi Sanjeev Mohanraj[1,†], Veerendra Dhyani[1], Angela Anna Baiju[1,2], Sihao Wang[1], Jiapeng Sun[3], Lin Zhou[4], Anna Paterova[1], Victor Leong[1], and Di Zhu[1,3,4,*]

[1]A*STAR Quantum Innovation Centre (Q.InC), Institute of Materials Research and Engineering (IMRE), Agency for Science, Technology and Research (A*STAR), Singapore 138634, Singapore
[2]Department of Physics, National University of Singapore, Singapore 117542, Singapore
[3]Department of Materials Science and Engineering, National University of Singapore, Singapore 117575, Singapore
[4]Centre for Quantum Technologies, National University of Singapore, Singapore 117543, Singapore
[†]These authors contributed equally
[*]e-mail: dizhu@nus.edu.sg

S1 Supplementary Table

Table S1. Comparison of normalized SHG conversion efficiencies in TFLN nanophotonic waveguides. *L*: waveguide length; η: normalized SHG efficiency; QPM: quasi-phase matching; MPM: modal phase matching; PPLN: periodically poled lithium niobate; LPLN: layer-poled lithium niobate.

**Ref**	**Mechanism**	***L*** (mm)	***η*** (%W^{-1}cm^{-2})
Ref[1]	QPM (PPLN)	21	2154
Ref[2]	QPM (PPLN)	6	3802
Ref[3]	QPM (PPLN)	6	3061
Ref[4]	QPM (PPLN)	5.7	975
Ref[5]	QPM (PPLN)	5	3757
Ref[6]	QPM (PPLN)	5	3256
Ref[7]	QPM (PPLN)	4	2600
Ref[8]	QPM (PPLN)	4	1735
Ref[9]	QPM (PPLN)	3	684
Ref[10]	QPM (PPLN)	1	1900
Ref[11]	QPM (PPLN)	0.6	4600
Ref[12]	QPM (Periodic groove)	0.5	6.8
Ref[12]	MPM	1	41
Ref[13]	MPM	1	26
Ref[14]	MPM (dual-layer)	1.2	5540
This work	MPM (LPLN)	2.5	4615

S2 Supplementary Figures

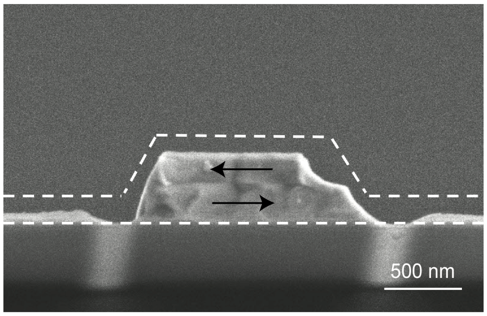

Figure S1. Scanning electron micrograph of the LPLN waveguide cross-section after intentional anisotropic wet etching. A clear boundary between the two domains and the discontinuity of their sidewalls can be observed, indicating layer-wise inverse polarities. The white dashed line marks the original LPLN waveguide cross-section before wet etching.

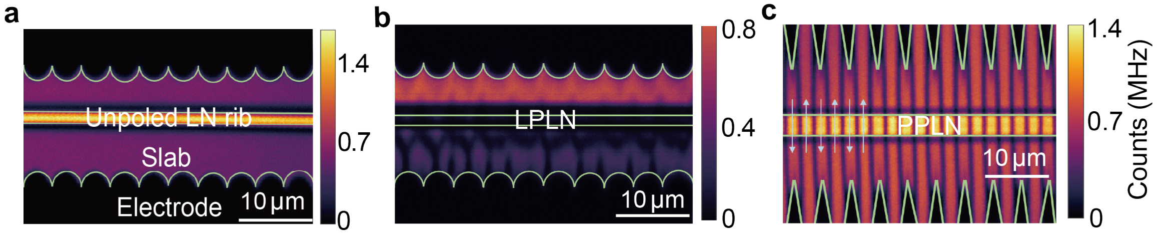

Figure S2. Laser scanning SHG microscopy of a LPLN waveguide (top-view). The dark areas, where the electrodes locate, do not produce SH light, because the metal does not exhibit second-order nonlinearity. Before poling (**a**), both the LN slab and the LN rib show homogeneous SH intensity. The thicker (600 nm) LN rib shows stronger SH intensity than that in the thinner LN slab (200 nm). A pair of dark lines are seen on both sides of the LN rib due to the angled sidewall, which diffracts and scatters SH light away from the objective collection angle. During poling, the polarity inversion starts from the bottom electrode, where we apply positive voltage. The SH intensity near the bottom electrode is not homogeneous because of the nonuniform electric field from the ripple-shaped electrode. The electric field gradually merges and tends to be uniform near the LN rib, so that the SH intensity of the LPLN waveguide and the upper slab is more homogeneous. The LPLN waveguide becomes dark after poling (**b**) due to partial poling (Fig. 1**d** in main text). The SH signals generated from the two layers are out of phase and destructively interfere, resulting in a reduced SH intensity. We notice that the SH intensities near the top (ground) and bottom electrodes (positive) are different. This is probably due to certain partial poling near the bottom electrode, but the exact reason is still unclear and needs further investigation. The SH intensity in the slab near the top electrode becomes slightly higher after poling. This could be due to counterpropagation phase matching from slight partial poling, which is reported in Ref.[15]. **c**, Laser-scanning SHG microscopy of a PPLN waveguide as a reference. Clear boundary lines are observed between fully poled, inversely oriented adjacent domains due to destructive interference of the SH signals.

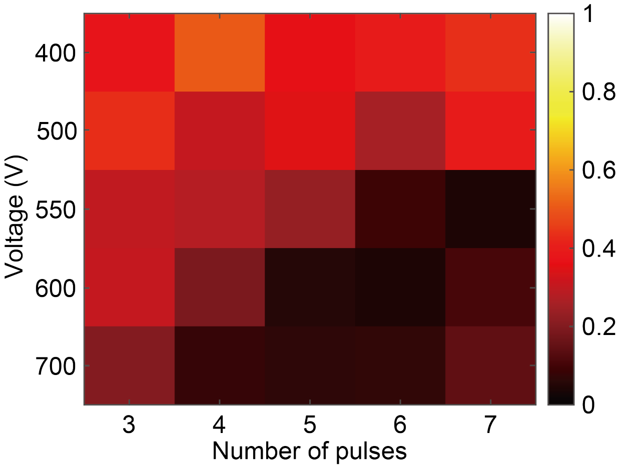

Figure S3. Relation between poling depth and poling conditions (poling voltage and number of pulses). Instead of directly visualizing each device's cross-section, we use the count rates from the laser-scanning SHG imaging (see Fig. 1**f** in main text) to infer the poling depth. Here, the colorbar is the ratio between the SHG signals in the poled and unpoled waveguides. A high ratio indicates weak spatial symmetry breaking in the poled waveguides (i.e., close to being completely unpoled or fully poled), and a low ratio indicates strong layer-wise symmetry breaking (i.e., partially poled) due to the destructive interference between the two inversely polarized layers. There is a reasonable margin for the selection of voltage and number of pulses to achieve the desired partial poling. The poling is performed after waveguide etching and at room temperature. We choose 6 pulses with 600 V voltage for the final device fabrication. Noted that it is also nontrivial to get perfect LPLN. It still requires optimized processes, for example, elevated temperature, structured poling electrodes, and multi-pulse with specific voltage range. However, our experimental results (Fig. S3, Fig. 2**c**-2**e**) suggest relatively improved fabrication tolerance including poling voltage and pulse number, and reduced sensitivity to waveguide geometry and temperature, as compared to PPLN.

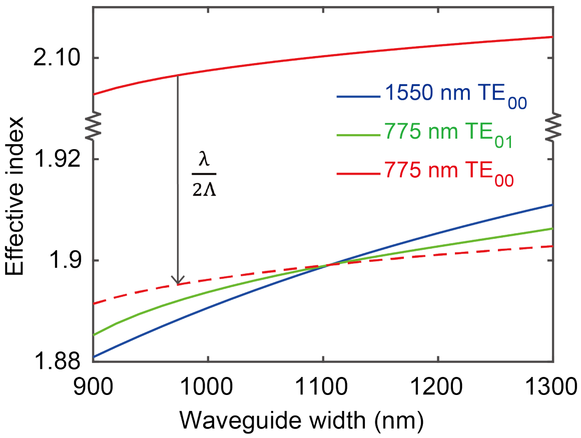

Figure S4. Simulation of effective indices of the TE_{00} mode at 1550 nm (blue), the TE_{00} mode at 775 nm (red), and the TE_{01} modes at 775 nm (green) as a function of LN waveguide width. As the waveguide width increases, the effective index of the FH TE_{00} mode exhibits a slower rate of increase compared to that of the SH TE_{01} mode. The intersection point at $\sim$1100 nm between the blue and green curves signifies the phase-matching point for MPM. A larger slope difference between the FH TE_{00} and SH TE_{00} modes is seen compared to that between FH TE_{00} and SH TE_{01} modes, indicating that QPM in PPLN is more sensitive than MPM in LPLN in terms of the waveguide width variation. Here, the $\lambda/2\Lambda$ shift indicates momentum matching offered by QPM in PPLN.

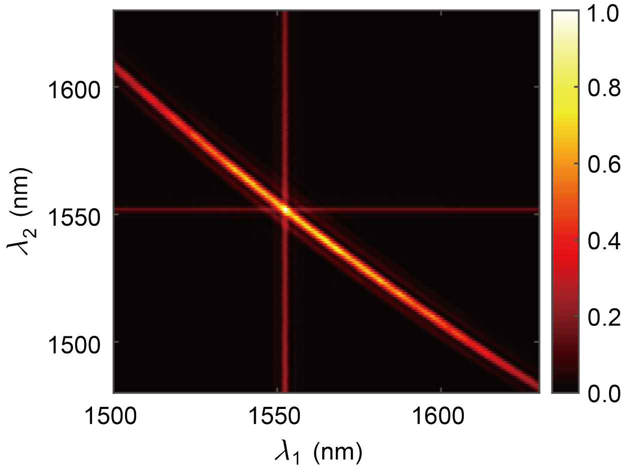

Figure S5. Sum-frequency generation (SFG) phase-matching function from 1480 nm to 1630 nm, measured by sweeping two telecom cw lasers and measuring the generated SFG power. The diagonal slope follows the energy conservation line ($1/\lambda_1 + 1/\lambda_2 = 1/\lambda_{SHG}$), allowing broadband SPDC.

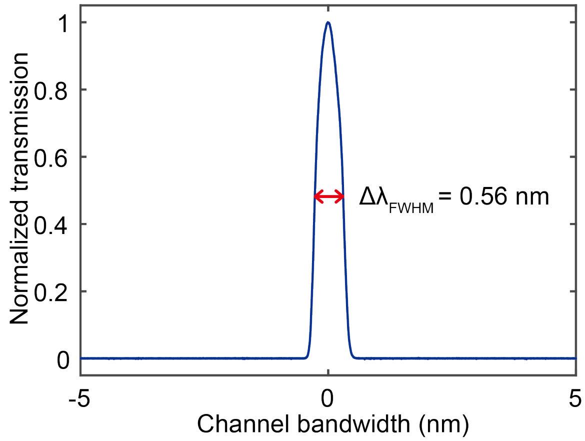

Figure S6. Measured transmission spectrum of the signal/idler channel used in the photon-pair measurement, showing a FWHM bandwidth of 0.56 nm. This bandwidth is used to calculate the SPDC brightness.

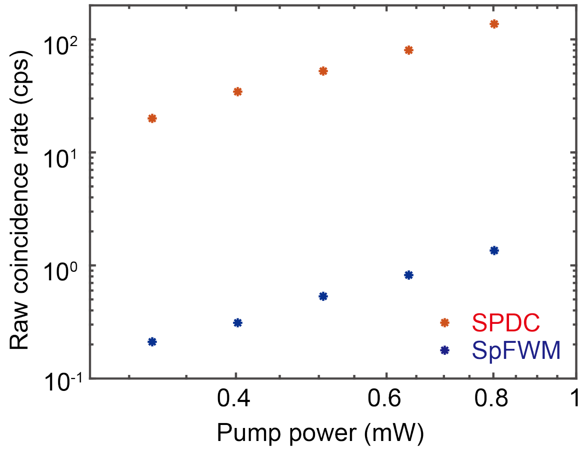

Figure S7. Measured off-chip, raw photon-pair generation rates from SPDC (red) and SpFWM (blue) as a function of on-chip pump power. The PGR from SPDC is about two orders of magnitude higher than that from SpFWM.

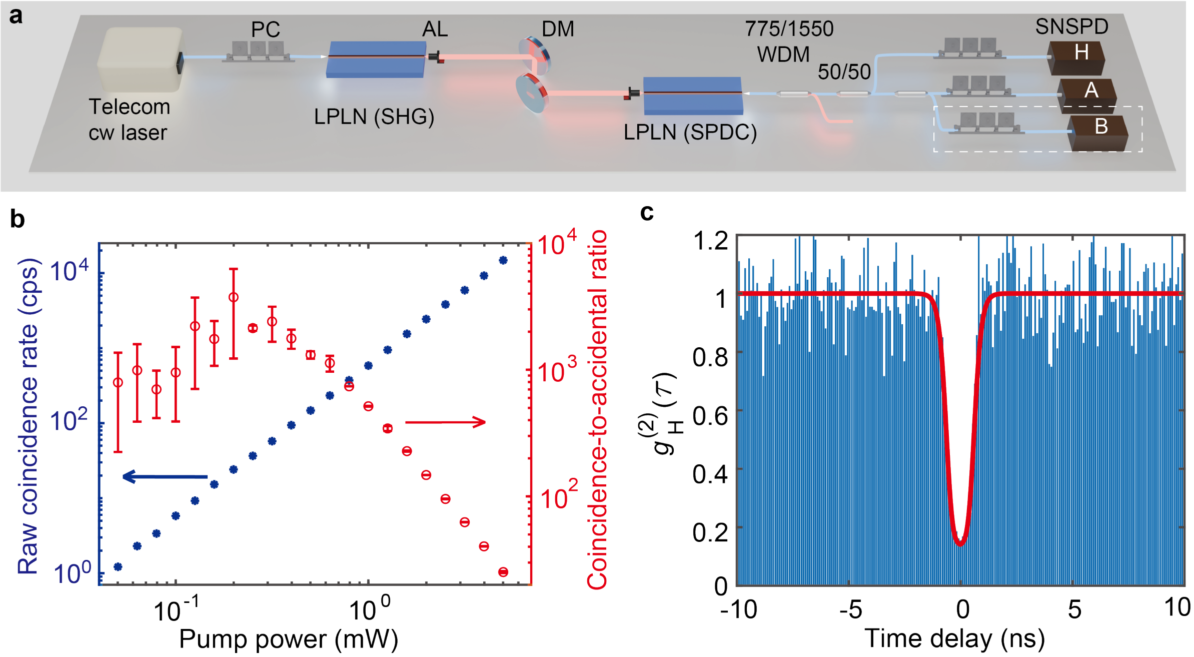

Figure S8. Photon-pair generation with two LPLN chips, one for SHG and the other for SPDC. a, Experimental setup. The SH light, generated in the first chip, is coupled out from the first chip and then into the second chip through a pair of aspheric lenses (AL). The telecom pump light is filtered in between using dichroic mirrors (DM). SPDC photon pairs generated in the second chip are coupled out using a lensed fiber, and the SH light is filtered out through a 1550 nm/775 nm WDM. Signal and idler photons are separated using a 50/50 beamsplitter and sent to SNSPDs for coincidence counting with H and A, and heralded second-order correlation function measurement with H, A, and B. **b**, Off-chip, raw measured photon-pair generation rate (blue) and CAR (red) versus on-chip pump power. Referencing to the 775 nm pump (from the first LPLN chip), we estimate the SPDC generation efficiency to be 4.2 GHz mW^{-1}. The actual on-chip SPDC rate may be underestimated due to the limited detection bandwidth (WDM, coupler, SNSPD, etc.). Moreover, high-order mode coupling is sensitive, and other modes can be excited in the second chip, which does not contribute to the SPDC generation due to phase mismatch. **c** Measured (blue) and fitted (red) heralded second-order correlation function versus time delay at a pump power of 5.0 mW, and it is measured to be 0.014 at zero time delay, indicating the measurements are operated in the single-photon regime.

S3 Scaling laws for cascaded SHG and SPDC process

In this section, we use a simplified classical model to derive the scaling laws for the cascaded SHG-SPDC photon-pair generation process. We note that this model does not capture the quantum nature of SPDC and is not sufficient to predict the absolute photon-pair generation rate or dynamics. In a cascaded SHG and non-degenerate SPDC process based on MPM without phase mismatch, the coupled amplitude differential equation can be expressed as

$$\frac{\partial A_1}{\partial z} = i\frac{\omega_1^2}{c^2 k_1} d_{\mathrm{eff}} A_1^*(z) A_2(z), \tag{S1}$$

$$\frac{\partial A_2}{\partial z} = i\frac{\omega_2^2}{c^2 k_2} d_{\mathrm{eff}} A_1(z) A_1(z) + i\frac{\omega_2^2}{c^2 k_2} d_{\mathrm{eff}} A_{\mathrm{s}}(z) A_{\mathrm{i}}(z), \tag{S2}$$

$$\frac{\partial A_{\mathrm{s}}}{\partial z} = i\frac{\omega_{\mathrm{s}}^2}{c^2 k_{\mathrm{s}}} d_{\mathrm{eff}} A_{\mathrm{i}}^*(z) A_2(z), \tag{S3}$$

$$\frac{\partial A_{\mathrm{i}}}{\partial z} = i\frac{\omega_{\mathrm{i}}^2}{c^2 k_{\mathrm{i}}} d_{\mathrm{eff}} A_{\mathrm{s}}^*(z) A_2(z), \tag{S4}$$

where ω and k are frequency and wavevector, respectively, and the indices 1, 2, s, and i indicate the parameters at FH, SH, signal and idler frequencies, respectively. Following energy conservation, we have $\omega_2 = 2\omega_1$ and $\omega_2 = \omega_{\mathrm{i}} + \omega_{\mathrm{s}}$. Furthermore, we assume the FH pump is in a non-depletion regime, and the FH and SH fields are much stronger than the signal and idler fields. Hence, A_1 can be treated as a constant, and Eq. S2 is simplified to

$$\frac{\partial A_2}{\partial z} = i\frac{\omega_2^2}{c^2 k_2} d_{\mathrm{eff}} A_1 A_1. \tag{S5}$$

Combining Equations S3-S5, we have

$$\frac{\partial^2 A_{\mathrm{s}}}{\partial z^2} - \frac{1}{z}\frac{\partial A_{\mathrm{s}}}{\partial z} - \frac{z^2}{g^4} A_{\mathrm{s}} = 0, \tag{S6}$$

where $g = \frac{(k_1^2 k_{\mathrm{i}} k_{\mathrm{s}})^{1/4} c^2}{(\omega_{\mathrm{s}}\omega_{\mathrm{i}})^{1/2}\omega_2 d_{\mathrm{eff}} A_1}$. Eq. S6 has a general solution in the form of

$$A_{\mathrm{s}}(z) \propto C_1 \sinh(z^2/2g^2) + C_2 \cosh(z^2/2g^2) \propto z^2/2g^2 + \mathscr{O}(z^4), \tag{S7}$$

where C_1 and C_2 are constants and the last approximation assumes $z \ll g$. For a short waveguide of length L, we can expect the SPDC rate in the cascaded SHG-SPDC process to scale as $P_{\mathrm{s}} \propto P_1^2 L^4$, where $P_{\mathrm{s}} = |A_{\mathrm{s}}|^2$ and $P_1 = |A_1|^2$. The quadratic relation between the photon-pair generation rate and the pump power agrees well with the measurement in Fig. 3**c**. The quartic relation between the signal power and the propagation length indicates that increasing the waveguide length is an effective means to further increase the pair generation efficiency. As a comparison, the photon-pair generation rate in standard SPDC with SH pump scales as $\propto P_{\mathrm{pump}} L^2$, and that in SpFWM with telecom pump scales as $\propto P_{\mathrm{pump}}^2 L^2$.

Supplementary information accompanies the manuscript on the Light: Science & Applications website (http://www.nature.com/lsa)

References

1. Chen, P.-K. *et al.* Adapted poling to break the nonlinear efficiency limit in nanophotonic lithium niobate waveguides. *Nature Nanotechnology* **19**, 44–50 (2024).
2. Li, X. *et al.* Advancing large-scale thin-film ppln nonlinear photonics with segmented tunable micro-heaters. *Photonics Research* **12**, 1703–1708 (2024).
3. Niu, Y. *et al.* Optimizing the efficiency of a periodically poled lnoi waveguide using in situ monitoring of the ferroelectric domains. *Applied Physics Letters* **116** (2020).
4. Fang, X.-X., Wang, L. & Lu, H. Efficient generation of broadband photon pairs in shallow-etched lithium niobate nanowaveguides. *Optics Express* **32**, 22945–22954 (2024).
5. Zhao, J. *et al.* Shallow-etched thin-film lithium niobate waveguides for highly-efficient second-harmonic generation. *Optics Express* **28**, 19669–19682 (2020).
6. Liu, X. *et al.* Ultra-broadband and low-loss edge coupler for highly efficient second harmonic generation in thin-film lithium niobate. *Advanced Photonics Nexus* **1**, 016001–016001 (2022).

7. Wang, C. *et al.* Ultrahigh-efficiency wavelength conversion in nanophotonic periodically poled lithium niobate waveguides. *Optica* **5**, 1438–1441 (2018).

8. Zhang, L. *et al.* Second-harmonic and cascaded third-harmonic generation in generalized quasiperiodic poled lithium niobate waveguides. *Optics Letters* **48**, 1906–1909 (2023).

9. Zhang, H., Li, Q., Zhu, H., Cai, L. & Hu, H. Second harmonic generation by quasi-phase matching in a lithium niobate thin film. *Optical Materials Express* **12**, 2252–2259 (2022).

10. Chen, J.-Y. *et al.* Efficient and highly tunable second-harmonic generation in z-cut periodically poled lithium niobate nanowaveguides. *Optics Letters* **45**, 3789–3792 (2020).

11. Rao, A. *et al.* Actively-monitored periodic-poling in thin-film lithium niobate photonic waveguides with ultrahigh nonlinear conversion efficiency of 4600% w- 1 cm- 2. *Optics express* **27**, 25920–25930 (2019).

12. Wang, C. *et al.* Second harmonic generation in nano-structured thin-film lithium niobate waveguides. *Optics Express* **25**, 6963–6973 (2017).

13. Chen, J.-Y., Sua, Y. M., Fan, H. & Huang, Y.-P. Modal phase matched lithium niobate nanocircuits for integrated nonlinear photonics. *OSA Continuum* **1**, 229–242 (2018).

14. Wang, L., Zhang, X. & Chen, F. Efficient second harmonic generation in a reverse-polarization dual-layer crystalline thin film nanophotonic waveguide. *Laser & Photonics Reviews* **15**, 2100409 (2021).

15. Ruesing, M., Zhao, J. & Mookherjea, S. Second harmonic microscopy of poled x-cut thin film lithium niobate: Understanding the contrast mechanism. *Journal of Applied Physics* **126** (2019).
